# Implementation of an intervention to scale up coverage of pneumonia management in children younger than 5 years in a north Indian district: protocol for a quasi-experimental, mixed-methods, pre-post implementation study

**DOI:** 10.1136/bmjopen-2024-093705

**Published:** 2025-07-07

**Authors:** Barsha Gadapani Pathak, Yasir Bin Nisar, Tarun Madhur, Naveen Garg, Shamim Qazi, Sarmila Mazumder

**Affiliations:** 1 Society for Applied Studies, New Delhi, India; 2 Faculty of Medicine, University of Bergen, Bergen, Norway; 3 Department of Maternal, Newborn, Child and Adolescent Health and Ageing, World Health Organization, Geneve, Switzerland; 4 Palwal District Health Authority, Palwal, Haryana, India; 5 Independent Consultant Paediatrician, Geneva, Switzerland

**Keywords:** Implementation Science, INFECTIOUS DISEASES, Community child health, QUALITATIVE RESEARCH, Child, Public health

## Abstract

**Abstract:**

**Introduction:**

The National Family Health Survey-5 has reported an under-five mortality rate of 41.9 per 1000 live births in India. Pneumonia, one of the leading causes of under-five mortality, contributes substantially to this figure. The Indian government has made efforts through multiple national programmes, but pneumonia-specific mortality remains high. The Government of India revised their Childhood Pneumonia Management Guidelines in 2019 to improve under-five pneumonia prevention and management. This implementation study aims to achieve a high population-based coverage of pneumonia treatment for under 5 yearold children in the Palwal district of India.

**Method and analysis:**

This implementation study uses a quasi-experimental pre-post design and a mixed-methods approach, conducted in three phases: (i) formative research, (ii) model optimisation through iterative testing in a learning block and (iii) scale-up and concurrent evaluation. The study is set in Palwal district, Haryana, and the primary catchment/study area will be the Health and Wellness Centres, the most accessible public health facilities for the community. Approximately 4167 households will be surveyed to capture ~2400 under-five children, among whom about 120 pneumonia cases (based on an estimated 5% prevalence) will be included in the analysis of treatment coverage and outcomes. Quantitative data will be analysed using descriptive statistics and generalised linear models, while qualitative data from focus group discussions and in-depth interviews will be thematically analysed using NVivo software.

**Ethics and dissemination:**

Ethical approval was granted by the ethical committees of the Society for Applied Studies (ERC/IR Pneumonia/2021), the Regional Ethics Committee of Western Norway (2022/531608) and the WHO(ERC.0003652). Additionally, this study has obtained the Government of Haryana state (Memo no. HSHRC/2022/505) and Health Ministry steering committee (approval date: 19 Dec 2022, proposal id 2022–17596) approvals. Informed consent will be obtained from all participants, including caregivers and healthcare workers, prior to data collection. Dissemination meetings in the study country will share results with stakeholders, including Ministry of Health officials, health managers, families of under-five children, community leaders and academia, to discuss national health programme implications. Results will also be shared regionally and globally, with publications and presentations encouraged in national and international forums.

**Study registration:**

Clinical Trials Registry – India, CTRI/2021/03/031622.

**STRENGTHS AND LIMITATIONS OF THIS STUDY**

- Uses a quasi-experimental pre-post design with mixed-methods to capture both outcome and process-level insights.
- Follows an iterative, adaptive implementation cycle that incorporates continuous learning and real-time strategy refinement.
- Employs systematic household sampling and Probability Proportional to Size methods to ensure representative quantitative data collection.
- Uses a broad operational definition of pneumonia based on caregiver-reported signs, which may reduce specificity and lead to misclassification.
- Relies on self-reported data for key indicators, introducing potential recall and reporting biases.

## Introduction

 The National Family Health Survey-5 (NFHS-5), conducted from 2019 to 2021, reported an under-five mortality rate of 41.9 per 1000 live births in India.^1^ Data from national surveys between 2016 and 2018 revealed that the top three causes of under-five deaths in India were lower respiratory infections (17.9%), neonatal preterm birth (15.6%) and other neonatal disorders (14.3%).^2^ Among infectious diseases, pneumonia is the primary contributor to under-five mortality, accounting for 15%.^3^ There are estimated 30 million annual cases of acute respiratory illness (ARI), including 3 million cases of severe pneumonia. These statistics highlight the critical public health concern posed by under-five pneumonia and its substantial impact on India’s goal to reduce under-five mortality to 23 per 1000 live births by 2025.^4^

Over the years, the Indian government has consistently supported pneumonia control measures through multiple national programmes, such as Home-based Newborn Care (HBNC), Home-based Young Child Care, Integrated Management of Neonatal and Childhood Illness, Facility-based Integrated Management of Neonatal and Childhood Illness, Poshan Abhiyan, Mother’s Absolute Affection programme, Universal Immunisation Programme/Mission. Indra Dhanush, Vitamin A supplementation, Infant and Young Child feeding programme and Facility-based Newborn Care, to address childhood infections prevention and enhance under-five infection management.^4^ Despite these efforts, India still witnesses an annual pneumonia-specific mortality of 0.14 million.^4^ Only 22% of caretakers seek appropriate care for acute respiratory infections due to factors like limited awareness, financial constraints, healthcare accessibility issues, reliance on home remedies and insufficient community involvement.^5^ Front-line health workers also face challenges, lacking the necessary skills and resources.^6^ Evidence from previous studies in India indicates that gender disparities exist in healthcare-seeking behaviour, with girls often receiving delayed or inadequate treatment for childhood illnesses, including pneumonia, necessitating a focused effort to promote equitable care through targeted strategies for female children.^7^ The primary challenge lies in the effective implementation of these well-intentioned national guidelines and programmes.^4^

Considering the substantial burden and the pressing need to intensify under-five pneumonia prevention and management efforts, the Government of India (GoI) launched the Childhood Pneumonia Management Guidelines in 2019.^4^ These guidelines represent a comprehensive shift from previous fragmented efforts by emphasising structured case management pathways across all levels of the health system. Key features of the CPMG include standardised management using oral amoxicillin and injectable gentamycin for pneumonia and possible serious bacterial infections (PSBI); use of pulse oximeters to assess severity, pre-referral treatment and referral mechanisms, integration of pneumonia indicators into the Health Management Information System (HMIS) and guidance for health system strengthening, including staff capacity building, community engagement and logistics support. In pursuit of ensuring the programme’s visibility and long-term sustainability, the Social Awareness and Actions to Neutralise Pneumonia Successfully (SAANS) campaign was initiated, aligning with the Protect, Prevent and Treat interventions for childhood pneumonia.^4 8^ This annual campaign runs from November 12th (World Pneumonia Day) to February 28th and aims to raise awareness among caregivers and communities about pneumonia prevention, recognition and treatment, which may eventually reduce the pneumonia burden.^4^ It mobilises Accredited Social Health Activists (ASHAs) and other front-line workers to conduct community outreach, home visits and behaviour change communication while promoting increased demand for pneumonia services. The strategic vision revolves around the utilisation of Health and Wellness Centres (HWCs), established under the Ayushman Bharat Yojana, as central nodes for early identification, treatment and timely referral of severe cases of under-five childhood pneumonia to tertiary healthcare facilities for life-saving interventions.^4^ Despite these advances, the full-scale implementation of the CPMG and SAANS initiatives has been limited in several states due to health system disruptions, particularly during the COVID-19 pandemic. Additionally, effective implementation of the CPMG hinges on several elements: accurate community-level case identification, trained providers, well-equipped health facilities and functional referral systems. Previous studies in India have shown that frontline workers often lack the required knowledge and confidence to follow pneumonia management protocols, and facilities frequently struggle with infrastructure and medicine shortages.^9^,^11^

Considering the above context, this implementation research was conceptualised to optimise and evaluate the delivery of the GoI’s Childhood Pneumonia Management Guidelines^4^ in a resource-constrained setting. The study focuses on achieving high population-based treatment coverage, defined as 80% or more under-five children receiving the full recommended treatment while aligning with and strengthening existing public health infrastructure. This study specifically aims to address several of these persistent barriers such as delayed care-seeking, low treatment compliance, inadequate provider capacity and limited adherence to national pneumonia management protocols, through a locally adapted implementation strategy. The research will identify practical implementation barriers and develop adaptable strategies that can inform scale-up efforts in similar settings across India.

### Research question and study objectives

#### Research question

How does an optimised implementation model influence the appropriate coverage of pneumonia management among under-five children, and what clinical, behavioural and system-level factors shape treatment outcomes, adherence and gender-related care-seeking patterns?

#### Primary objective

To develop, refine, implement and evaluate an optimised, sustainable and scalable implementation model for improving population-based coverage of pneumonia management among under-five children, in alignment with the GoI’s Childhood Pneumonia Management Guidelines.^4^ The model aims to achieve ≥80% treatment coverage in the target population.

#### Secondary objectives

- To assess the proportion of children who recover after 7 days of treatment.
- To estimate the proportion of treatment failures among diagnosed pneumonia cases.
- To measure compliance with the recommended pneumonia treatment protocol.
- To determine the proportion of children who complied with hospital referral advice.
- To document the duration and treatment details of inpatient pneumonia cases.
- To explore the knowledge, perceptions and experiences of caregivers, healthcare providers and community health workers regarding pneumonia identification, referral and management.
- To identify barriers to and facilitators of equitable pneumonia treatment, particularly among girl children, in the context of the ‘Save the Girl Child’ initiative.

Additionally, the household level and facility level cost for management of the pneumonia cases before and after implementation of the guideline using the optimised model will also be collected by the research team.

## Methods and analysis

### Study design

This implementation study includes a mixed-method approach aiming to scale up the management of under-five pneumonia and uses a quasi-experimental pre-post design. Our approach involves a non-sequential, repeated, cyclical and iterative implementation process, characterised by a series of overlapping cycles that involves implementation, process learning with both quantitative and qualitative feedback and ongoing quantitative coverage evaluation.^12^ Regular meetings in collaboration with government officials will provide a platform for deliberation on the implementation progress, the insights acquired, the assessment of model performance and the continuous enhancement of the implementation process.^12^

### Implementation science framework

We will employ a flexible and adaptive strategy, actively engaging relevant stakeholders, thoroughly examining contextual factors, identifying implementation barriers and facilitators, allocating essential resources as needed and establishing a continuous monitoring system. These components collectively contribute to our aim of ensuring the success and sustainability of this guideline, embodying key elements of a robust implementation framework.^12^

### Operational definitions

In this, pneumonia is defined as the mother/caregiver reporting any episode of cough along with either fast breathing or difficult breathing or stridor (‘khar khar’ or ‘khad khad’) or chest indrawing or pneumonia (‘pasliyon ka chalna’) or any danger signs (eg, axillary temperature <35.5 or ≥37.5 °C or not able to feed or convulsion or movement only when stimulated or no movements at all among children aged less than 59 days and inability to breastfeed or drink or vomits everything or lethargy or reduced level of unconsciousness or convulsions among children ageing 2–59 months) in the last 28 days.^4^ In a low-resource setting like India, grassroots-level workers like ASHAs and caregivers, sometimes non-literate, are the ones reporting cases of pneumonia to the health facility. In this context, it is challenging to employ a definition of pneumonia that requires access to laboratory or radiological examinations. Considering the existing capacity of the health services, we need to employ a definition that is based on clinical signs that can be recognised by caretakers or ASHAs.^5^ Hence, the definition of under-five pneumonia, in this study, is broad (with low specificity) for operational reasons. Additionally, this operational definition has been employed in other studies which were conducted in similar settings in India.^5^

### Study setting

**Figure 1:**
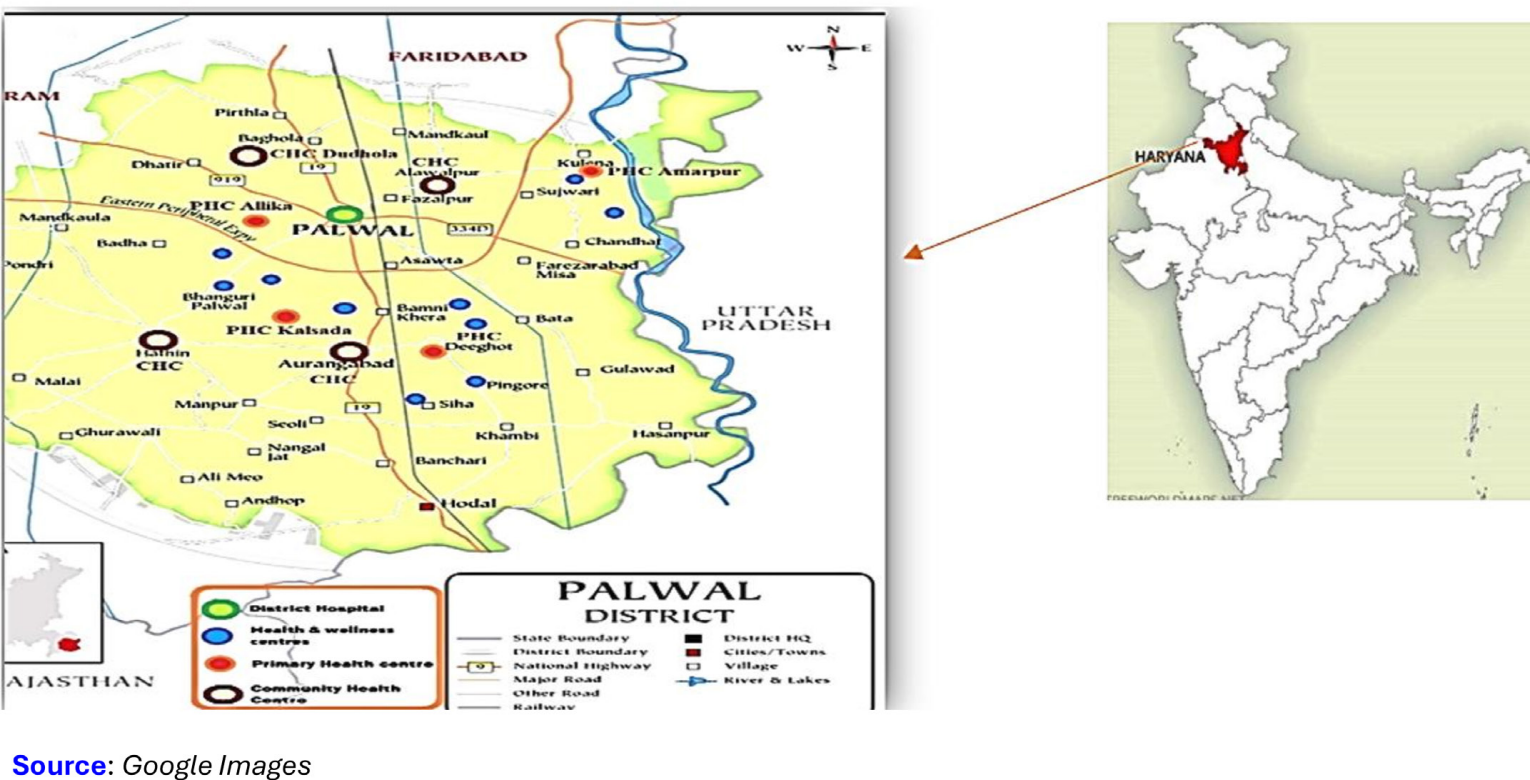
Map of the study area.

The study will be conducted in the Palwal district, located in the northern Indian state of Haryana, and covers an area of 1359 square kilometres, including three administrative divisions known as tehsils: Palwal, Hodal and Hathin^13^ (figure 1). It comprises 282 villages, 237 Gram Panchayats, one municipal council, two municipal committees, three subdivisions and four development blocks and is located approximately 80 km from the capital of India. This district serves a population of 1 273 665 through one District Hospital (DH), seven Community Health Centres (CHCs), 26 Primary Health Centres (PHCs), 49 functional HWCs and 1108 Anganwadi centres. The PHCs target to serve around 49 000 people each, with 10 subcenters catering to about 12 000 to 13 000 people each.^13^ The district has 37 medical officers at CHCs and PHCs, 176 medical officers and other healthcare professionals at the district hospital. The average literacy rate is 69.32%, and 12% of the population is under five years of age with an annual birth rate of 25 per 1000 population.^13 14^

The catchment area for our implementation research will be the HWCs of Palwal district. The HWCs were established under the Aayushman Bharat—Haryana Health Protection Mission, and currently, 49 are functional in the Palwal district to provide comprehensive primary healthcare services in proximity to residents’ homes.^14^

The district health authorities will determine the number of HWCs to be assessed from 49 functional centres and subsequently share the list with the research team. After the preliminary assessment, the research team will select ten HWCs for inclusion in the study (figure 1). We will exclude HWCs that lack appropriate infrastructure and human resources, as these are basic prerequisites for implementing the intervention. Specifically, HWCs must have a functional building, essential utilities such as electricity and water supply, and basic furnishings. Additionally, the presence of adequate human resources to deliver comprehensive primary healthcare services is critical. HWCs should also be operational on a regular basis. Facilities that do not meet these criteria would require significant upgrades, which may be time-consuming and not feasible within the study period. Among the eligible facilities, preference will be given to HWCs that are reasonably accessible (within approximately 11–12 km) from the DH to facilitate referral linkages and supervision.

Furthermore, in addition to the selected HWCs, the research team will conduct other facility assessments (PHCs, CHCs and DH) that cater to the population referred from these HWCs. The catchment area of this study will have an estimated population of 1,07,440, consisting of 42 villages and 9897 under-five populations.^13^

### Eligibility and inclusion criteria

The mothers/primary caregivers of the under-five children and healthcare workers in the facility and community within the catchment area will be included in this study.

### Sample size

**Figure 2:**
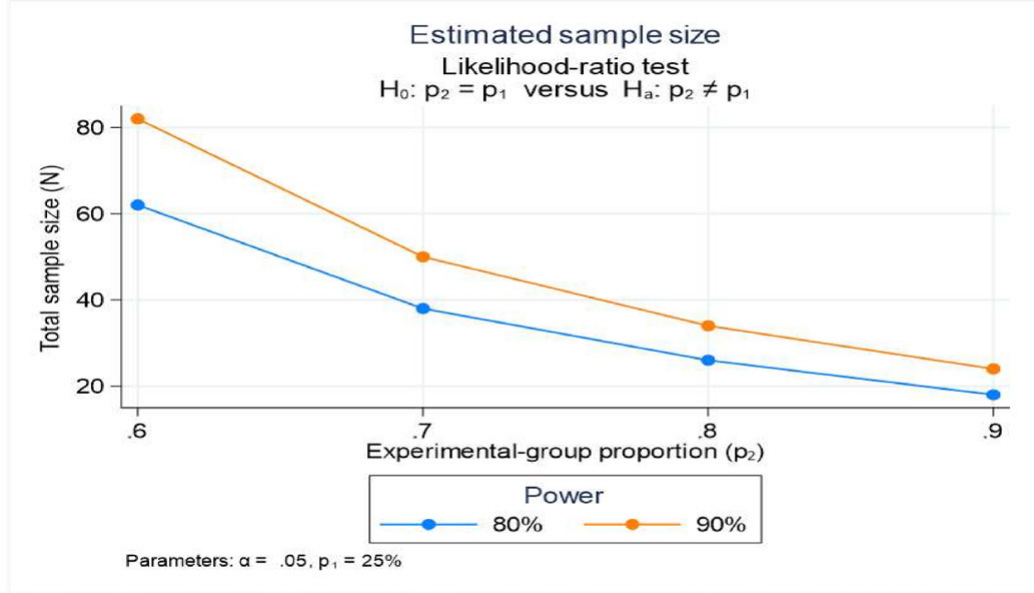
Sample size estimation.

A recent study in the same district found that the prevalence of pneumonia among under-five children in the last 2 weeks was around 5%.^15^ Currently, approximately 25% of under-five children seek appropriate care for ARI, indicating a programme coverage of around 25%.^5 16^ With the introduction of the new implementation strategy, we expect an increase in pneumonia treatment coverage by 80%. To account for potential non-response and to estimate 80% coverage of appropriate treatment at baseline and end-line surveys for under-five children with pneumonia cases (as per the operational definition), a conservative sample size of 120 is to be included. Additionally, based on these assumptions, the estimated sample size at a 5% level of significance for each survey (baseline and end-line), and a varied range of coverage is shown in figure 2.

The total number of children to be surveyed is calculated based on a prevalence rate of 5%, resulting in an estimated sample size of 2400.^5^ Considering the proportion of 5-year-olds in the population (12% based on census data) and the average household size (4.8 as per the NFHS 5 report for Haryana), the number of households to be covered is calculated as 4167 households.^17^ These households will be selected using Probability Proportional to Size (PPS) sampling methods from the defined study area served by the 10 HWCs.^18^ Since community-level healthcare workers maintain a list of under-five children in each household, systematic random sampling will be used to select children within the chosen households.

### Outcomes

The primary outcome of the study is to find the coverage of effective pneumonia management (ie, management done as per the childhood pneumonia management guidelines of India) from appropriate sources. Appropriate sources refer to government and/or private healthcare facilities like HWCs, PHCs, CHCs, DHs, private clinics/hospitals, etc, where treatment is provided by qualified healthcare practitioners. In contrast, non-appropriate sources include unlicensed practitioners, chemists, traditional or religious healers, and others who are non-registered medical healthcare workers. The secondary outcomes are total recovered cases after 7 days of treatment, treatment failure, compliance to management as recommended by healthcare providers, in-patient case duration and treatment provided, and gender-wise distribution of diagnosed, treated and complaint cases.

### Description and tracking process of study outcomes

The primary targeted outcome of this study is achieving ≥80% coverage of appropriate pneumonia management and will be assessed using a structured quantitative household survey tool. Three cross-sectional surveys (baseline, midline and endline) will be conducted with caregivers of under-five children who experienced pneumonia-like symptoms in the previous 28 days. These surveys will capture whether care was sought from appropriate sources and whether treatment received aligned with the national guidelines. The success of this research will be when at least 80% of these children:

- Received care from appropriate sources
- Were managed according to the recommended national protocols with ≥80% compliance to the prescribed treatment.

### Data collection for monitoring progress

To monitor progress towards the coverage target, both quantitative and qualitative data will be collected at community and facility levels:

- **Community-level quantitative data** (via household cross-sectional survey) will specifically include:
- **Facility-level data** will be collected at baseline (Phase-I), then bi-monthly (Phase-II and III) and finally at end-line through a cross-sectional survey. This will include:

The source of treatment for pneumonia, time to treatment-seeking after symptom onset, type of treatment received (with verification of medicines/injections where possible), compliance with treatment, reported treatment outcomes out-of-pocket expenses incurred.

- **Community-level qualitative data** will include caregivers’ knowledge of pneumonia signs and symptoms, care-seeking behaviour and reasons for provider preference, barriers and enablers to accessing appropriate care, experiences with service delivery and any suggestions to improve pneumonia care services.

- **Facility-level data** will be collected at baseline (Phase-I), then bi-monthly (Phase-II and III) and finally at endline through a cross-sectional survey. This will include the number of suspected pneumonia cases presenting at health facilities; number of cases diagnosed, treated or referral made as per guidelines; number of household (diagnosed cases of pneumonia) follow-ups conducted by ASHAs; availability of essential medicines and supplies; status of key equipment (eg, pulse oximeters, thermometers, weighing scales, stethoscopes); human resource availability; and training status of healthcare workers in pneumonia management.

These data streams will allow ongoing tracking of both outcome-level changes (ie, coverage and compliance) and process-level improvements (ie, facility readiness, staff capacity), helping inform adaptive changes to ensure progress towards the final targeted outcome.

### Intervention package

The intervention package will be aligned with the GoI guidelines, which include early identification of pneumonia cases through ASHAs and empowering families, prompt care-seeking from appropriate providers for pneumonia treatment and prompt referral of severe pneumonia cases with pre-referral treatment. At the outpatient department (OPD) of facilities (HWCs/PHCs/CHCs/DH), treatment providers need to be available, be competent and have access to appropriate logistics (equipment, regular supplies of medicines). Pulse oximeters will be provided at the OPDs of these facilities to assess oxygen saturation for improved assessment/evaluation of severe cases requiring referral.

For children, (0–59 days) with PSBI, a prereferral dose of gentamicin and amoxicillin will be provided by the health workers at the HWCs, and then cases are referred to higher facilities. But if a referral is not possible/denied, the package also aims to provide treatment to children aged 0 to 59 days in the outpatient department of primary-level care. In cases where caregivers are unable to bring their infants to the health facilities for the full course of injection, gentamycin and oral amoxicillin for 7 days will be provided by the auxiliary nurse midwives to cases (0–59 days) at home.^4 19 20^

In discussions with the government, strategies will be finalised to promote treatment in female children, such as providing additional incentives to ASHAs for referring or accompanying girl children for pneumonia treatment, free transportation for girl children to facilitate referral for outpatient treatment and providing free food or compensating daily wages lost due to hospital admissions.

At the post-facility level, ASHAs will be rewarded (with government consensus) for ensuring full compliance with the treatment course in female children. The decision to address the appropriate treatment of pneumonia in the private sector, where many children with pneumonia seek care, by involving private providers in the discussion with the government partners, will be based on the formative research findings. Appropriate use of bronchodilators in children with wheeze at health facilities and identification of conditions mimicking pneumonia for rational therapy will also be included. Finally, recognition of cases that do not have pneumonia and do not require antibiotics but may benefit from supportive treatment.

**Figure 3:**
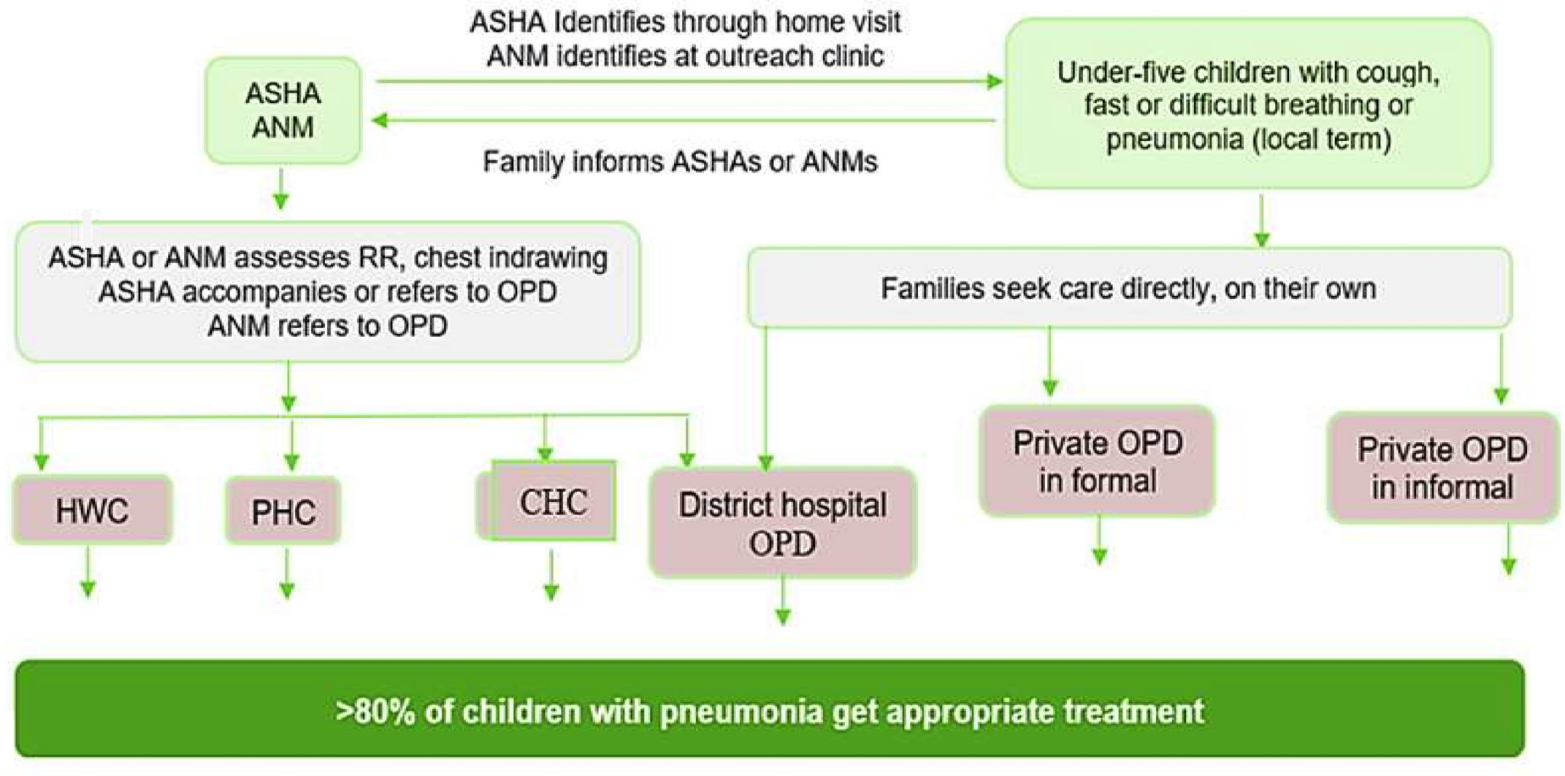
Conceptual framework for increasing coverage of pneumonia treatment. ANM, auxiliary nurse midwife; ASHA, accredited social health activist; CHC, Community Health Centre; HWC, Health and Wellness Centre; OPD, outpatient department; PHC, primary healthcare; RR, respiratory rate.

In the process, all collateral programmes supporting pneumonia management will be strengthened like Janani Shishu Suraksha Karayakram (for free transportation, medicines and diagnostics test availability), Comprehensive Primary Healthcare (strengthening care at HWCs) and HBNC (improving the quality of care by ASHAs). All the activities will be conducted with deep government engagement. The conceptual framework of implementing this intervention is summarised in figure 3.

### Research teams

There will be three teams: (i) the Implementation Support Team (IST), (ii) the Programme Learning Team (PLT) and (iii) the Outcome Measurement Team (OMT).

The IST will provide technical support to government partners and assist in programme implementation. They will facilitate government staff training in the study area, identify process gaps for refinement and develop tools and job aids. Additionally, the IST will support community awareness activities and data collection from ASHA records and healthcare facilities.

The PLT, composed of qualitative researchers, will assess fidelity and compliance through methods like focus group discussions (FDGs), in-depth interviews (IDIs) and observations. They will provide prompt feedback to the IST and deliver weekly reports.

The OMT will focus on measuring primary and secondary outcomes. They will conduct baseline and endline cross-sectional surveys in randomly selected households within the study area, assessing population-based programme coverage using a randomised sample selection method.

### Implementation strategy

**Figure 4:**
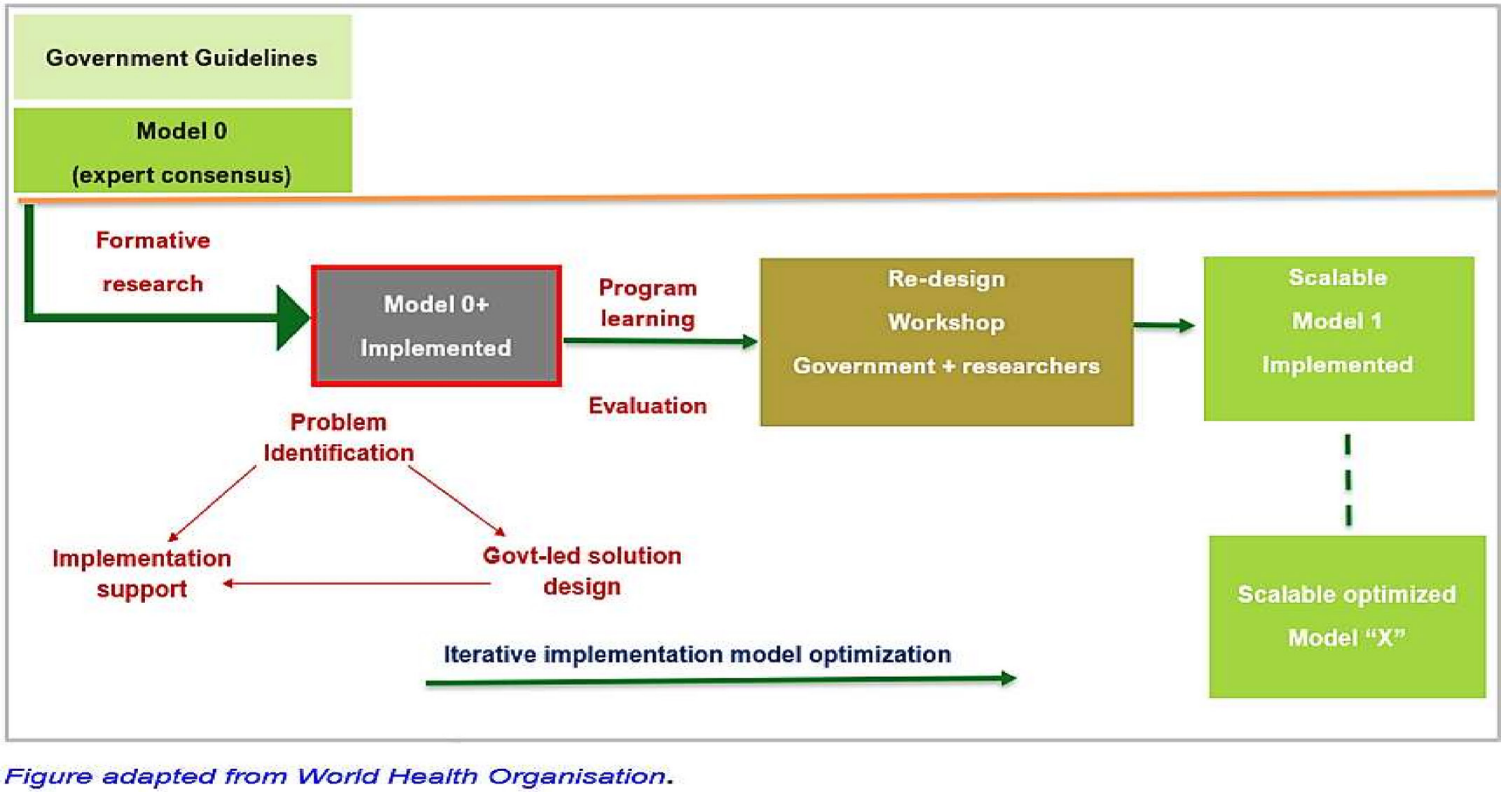
Iterative cycles of model optimisation.

The implementation plan for promoting the treatment of pneumonia in under-five children will follow a systematic approach involving formative research, engagement of key stakeholders, model development, implementation and evaluation (figure 4). The study will consider the GoI childhood pneumonia guidelines and develop model 0 through expert consensus. IDIs and FDGs will be conducted to gain a comprehensive understanding of stakeholders’ perspectives, knowledge and practices related to pneumonia management. For the implementation of guidelines, the key stakeholders, including mothers/primary caregivers, healthcare workers, community health workers and government partners, will be identified and actively engaged in the study catchment area.

The formative research (phase-I) findings will be analysed to identify pneumonia management barriers and facilitators, and a Model 0+implementation strategy will be developed. This model will include strategies to create caregiver awareness, encourage care-seeking at appropriate health facilities, improve community health workers’ skills in case identification and referrals and provide training and capacity building for other healthcare workers to enhance early identification and treatment of pneumonia cases. Workshops will be conducted with the research team and government partners to refine and finalise Model 0+ for local alignment and needs.

Following the development of Model 0+ will be implemented in a learning block comprising four randomly selected HWCs, with the close collaboration of government partners, to provide initial handholding and support for implementation. Monitoring and evaluation of process indicators such as access, utilisation, compliance, adherence and fidelity to childhood pneumonia management guidelines, as well as health facility components like infrastructure, human resources, delivery, availability of medicines and supplies, and documentation, will be conducted. Evaluation data will be analysed and shared with the government implementation team monthly to guide decision-making. Any inconsistencies identified between the evaluation data and existing system data will be addressed through corrective actions.

The research findings from the learning block will be used to enhance Model 0+, leading to the development of Model 1. Subsequently, a series of rapid cycles involving concurrent implementation, monitoring and strategy refinement will be conducted in the remaining six HWCs. These iterations will take place in multiple co-design workshops, with significant involvement from government authorities. The iterative process will continue until an optimised and comprehensive strategy/model is achieved with the potential for achieving high coverage and quality of pneumonia management among under-five children in the study areas (figure 4). The model will be considered optimised when the healthcare providers can consistently recognise pneumonia signs and symptoms and manage cases in accordance with the national guidelines, with ≥80% fidelity among the suspected cases visiting the centres. Second, treatment compliance among under-five children reaches at least 80%, who have received this treatment from the health facilities.

Once the model is optimised, it will be scaled up to all ten HWCs in the study area. During this scale-up phase (estimated to last 7–8 months), we will continue to monitor both implementation barriers and quantitative indicators such as fidelity to the guidelines and treatment compliance. At the conclusion of the scale-up phase, an endline household survey will be conducted using PPS sampling to identify 120 under-five children who had pneumonia symptoms in the past 28 days. These data will be compared with baseline measurements to assess changes in key outcome indicators, i.e. under-five pneumonia cases receiving management (as per the national guidelines) from the appropriate health facilities, which determines the coverage of appropriate pneumonia management.

The final findings will be shared with state- and district-level health authorities in Haryana. If the district government expresses interest and supports the approach, the model, having already been scaled up within the study area, may be adopted for implementation across the entire Palwal district, with continued technical support from the research team.

### Data management and data analysis

#### Quantitative data

The study’s data management will be handled by a data management centre (DMC) located in the study field office. The DMC will be using the Research Electronic Data Capture, which is a secure web application for building and managing clinical research data. It is a powerful and flexible tool for data management that can help streamline the research process and improve data quality. The quantitative data will be managed and reviewed by the respective team coordinators, and the data entry system will have inbuilt range and consistency checks. Cross-sectional survey data will be collected electronically and transferred in real-time to a local server at the DMC, where checks across forms and logical error checks will be run. Queries will be generated for the study team’s resolution, corrections will be incorporated into the cleaned data set and the tables for quality assurance will be generated at the Society for Applied Studies (SAS) office in Delhi daily.

Four types of data will be collected: (i) health systems data, (ii) routine patient data, (iii) cross-sectional household survey data and (iv) information on knowledge of pneumonia signs and symptoms and its management. Descriptive and inferential statistical analyses will be performed, including simple proportions and generalised linear models, using the univariate and multivariate regression analyses. The study will determine the prevalence of pneumonia, overall coverage of pneumonia treatment, compliance to treatment and treatment failure and identify predictors of treatment failure.

#### Qualitative data

Qualitative data will be collected to understand the experiences of healthcare providers and community health workers in treating pneumonia, as well as the perceptions of mothers and family members. Field notes will be recorded and transcribed from FGDs and IDIs. We will use the NVivo software package for data management and analysis. Data analysis will be conducted simultaneously with data collection, and relevant text will be coded daily. Codes will be descriptive or analytical, based on the research question, and grouped into themes and sub-themes. A framework analysis will be used, primarily a case and theme-based analysis. A matrix will display information to examine information across rows and down columns for theme development and to maintain context. Findings will be weighted by identifying key themes and estimating the number of times they appeared and the number of respondents who mentioned them.

### Study status

The study is currently ongoing and progressing through its planned phases. Phase I (Formative Research) was conducted between June and August 2023, during which contextual barriers, qualitative data collection from community, stakeholder perspectives and baseline data were collected. Phase II (Model Optimisation and Implementation in the learning block) commenced in November 2023 and concluded in May 2024, which involved iterative refinement of the implementation model through stakeholder engagement and real-time monitoring.

Phase III (Scale-up and Evaluation) began in June 2024 and will continue until May 2025. The endline household survey is scheduled to be conducted between February and April 2025, followed by qualitative data collection from the last week of April through May 2025. If the Government of Haryana decides to adopt and scale up the implementation model across the entire Palwal district, discussions on addressing barriers and implementation challenges will be integrated into monthly stakeholder review meetings. The study team will continue to provide technical assistance and support throughout this period, extending until the completion of the project (estimated June 2025).

### Patient and public involvement

Before the development of this protocol, we had a pilot phase which had extensive engagement within the research team, government stakeholders, community representatives and beneficiaries like the caregivers of under-five children and their families.

### Ethics and dissemination

This study will be conducted in accordance with the principles of Good Clinical Practice and ethical standards for implementation research. Ethical approval was granted by the ethical committees of the SAS (ERC/IR Pneumonia/2021), the Regional Ethics Committee of Western Norway (2022/531608) and the WHO(ERC.0003652). Additionally, this study has obtained the Government of Haryana state (Memo no. HSHRC/2022/505) and Health Ministry steering committee (approval date: 19 Dec 2022, proposal id 2022–17596) approvals. Before enrolment, all participants, including mothers or primary caregivers of under-five children and healthcare workers, will be provided with written informed consent forms in the local language. For non-literate participants, the consent form will be read aloud, and consent will be documented with a thumb impression in the presence of an impartial witness. Participation will be voluntary, and individuals may withdraw from the study at any time without any consequence. This protocol complies with the reporting guidelines of the Strengthening the Reporting of Observational Studies in Epidemiology and the Standard for Reporting Qualitative Research guidelines.^21 22^

The study places a high priority on protecting participant confidentiality. Data custody will be maintained by the data coordinator, with access granted only to the authorised data manager. All data collection devices and systems will be password protected. Real-time data will be securely transferred and stored on a cloud-based server, accessible only to authorised research personnel. Identifiable personal information (such as names, addresses or contact numbers) will be securely stored and separated from the analytical dataset. De-identified data may be shared with the WHO for further analysis.

Prior to the development of this protocol, a pilot phase involved extensive engagement with key stakeholders including government officials, community health workers and caregivers of under-five children to inform the design and implementation strategy.

Dissemination meetings will be held in India to share key findings with stakeholders, including officials from the Ministry of Health and Family Welfare, state and district health managers, community leaders and academic partners. Results will also be presented at regional and global forums. Manuscripts will be submitted for publication in peer-reviewed journals and findings will be shared at relevant national and international conferences to inform broader public health policy and programming.

## Discussion

Pneumonia remains the leading infectious disease-related cause of death among children under five in India, responsible for 15% of such fatalities. Achieving the National Health Policy’s goal of reducing under-five mortality to 23 per 1000 live births by 2025 necessitates a substantial reduction in pneumonia-related mortality to 3/1000 live births. This implementation research strives to develop a scalable strategy for pneumonia management, focusing on extensive population-based coverage of under-five pneumonia treatment recommended by the GoI guidelines. The study will comprise formative research, model optimisation and evaluation across the selected study area in Palwal district, Haryana, India, encompassing outpatient and inpatient pneumonia management.

The study’s optimised pneumonia management strategies, developed following implementation research principles, can be applied in similar low- and middle-income countries, enhancing coverage and reducing under-five pneumonia case fatality rates. Integrating key outcome indicators into the HMIS promotes accountability and sustainable scale-up. Furthermore, the research identifies system-level factors impacting healthcare system performance, allowing targeted improvements in areas like training, supply chain and healthcare worker payments, thereby strengthening overall coverage of pneumonia treatment.

In Indian and other low to middle-income settings, cluster-randomised controlled effectiveness trials have explored the management of uncomplicated pneumonia in infants aged 7 days to 59 months, employing community-level health workers. It reported that trained community-level workers like ASHAs can effectively manage the cases and are generally accepted in this role by the community.^23^ However, the Indian government chose not to employ this strategy due to concerns about ASHAs lacking the essential skills and educational qualifications for managing under-five illness. Consequently, the GoI’s guidelines focus on ASHAs primarily identifying pneumonia cases and referring them to the nearest HWCs for treatment. This implementation research aims to assess the practicality of implementing the guidelines and explore alternative strategies in collaboration with the government.

The proposed study exhibits multiple strengths, such as its concentration on a resource-constrained environment and its adherence to the core principles of implementation research. This entails addressing practical obstacles, formulating strategies to surmount these challenges and creating an optimised implementable model derived from acquired knowledge and insights. This model can subsequently be adapted to similar settings, allowing for broader scalability while considering contextual variations.

However, we must acknowledge that the study findings may be limited by the reliance on self-reported data from caretakers, which may be subject to recall bias. This study uses a broad operational definition of pneumonia that can be recognised by caretakers and community health workers, which may lead to an increase in false-positive cases reported to healthcare facilities. While the current study primarily concentrates on expanding the adoption of recommended interventions outlined in the guidelines, it is imperative to investigate the impact of the range of interventions as specified in the guidelines. This could be achieved through a hybrid type-II design, which falls beyond the purview of our present study.^24^ To achieve the desired power for outcomes, a large sample size will be required, which mandates extensive funding and resources.

This study underscores the importance of addressing healthcare-seeking behaviour among caretakers, improving the capacity of healthcare providers and increasing coverage of appropriate management to reduce pneumonia-related mortality in under-five children. Implementation research can help identify system-level factors that may impact the performance of the healthcare system in managing pneumonia cases and contribute to strengthening the capacity and effectiveness of the healthcare system in treating pneumonia.
