# Supplementary material for: Implementation of an intervention to scale up coverage of pneumonia management in children younger than 5 years in a north Indian district: protocol for a quasi-experimental, mixed-methods, pre-post implementation study
Source: BMJ Open. 2025 Jul 7;15(7):e093705. doi: 10.1136/bmjopen-2024-093705 (PMC12258345; doi:10.1136/bmjopen-2024-093705)
Supplement: Supplementary file 1 [file bmjopen-2024-093705.reviewer_comments.pdf]

PEER REVIEW HISTORY

BMJ Open publishes all reviews undertaken for accepted manuscripts. Reviewers are asked to complete a checklist review form and are provided with free text boxes to elaborate on their assessment. These free text comments are reproduced below.

ARTICLE DETAILS

Title (Provisional)

Implementation of an intervention to scale-up coverage of pneumonia management in children younger than 5 years in a North Indian district: protocol for a quasi-experimental, mixed-methods, pre-post implementation study

Authors

GADAPANI PATHAK, BARSHA; Nisar, Yasir Bin; Madhur, Tarun; Garg, Naveen; Qazi, Shamim; Mazumder, Sarmila

---

VERSION 1 - REVIEW

**Reviewer**	**1**
**Name**	**Gothankar, Jayashree**
**Affiliation**	**Bharati Vidyapeeth Deemed University Medical College Pune, Community Medicine**
**Date**	**27-Dec-2024**
**COI**	**None**

None

**Reviewer**	**2**
**Name**	**Nantanda, Rebecca**
**Affiliation**	**Makerere University, Lung Institute, Makerere College of Health Sciences**
**Date**	**07-Apr-2025**
**COI**	**None**

Summary

This implementation research study focuses on improvements in the implementation of strategies to reduce pneumonia mortality in India, focusing on community and primary care levels. The implementation is anchored on the Childhood Pneumonia Management Guidelines of 2019 developed by the government of India as a response to the persistently

high pneumonia morbidity and mortality among children less than 5 years of age. The study will use a quasi-experimental study design, and mixed (qualitative and quantitative) data collection methods will be used. It is a very important study that seeks to gather data on real-life situations and use it to develop a model that can be scaled up to other similar settings.

Comments

Background

The authors state that the pneumonia morbidity and mortality is still high, but the presumed factors, which will fully or partly be addressed by the current study are not clear.

It is not clear what is entailed in the Childhood Pneumonia Management Guidelines of 2019, and how different they are from all previous programs. A brief mention of how the previous programs were implemented, highlighting the gaps that will be closed by the current strategy should be mentioned.

The authors also need to provide some insights into the Social Awareness and Actions to Neutralize Pneumonia Successfully (SAANS) as this seems to be only in India.

The last paragraph of the background section focuses more on what will be done, which ideally should be under the implementation process. The details of what will be done under each proposed strategy (for example augmenting all supplementary programs on pneumonia) need to be provided.

The background and the rationale for focusing on the girl child is not provided. This needs to be included.

Study aim: The study aim is to develop a model, informed by the learnings from the implementation process. This is clear. However, the specific objective seems to be more like a desired/anticipated outcome/target rather than a research objective. This is also mentioned as a primary aim in the second last paragraph of the background section. The research question that the authors seek to answer are not stated or described.

Methods

Study design: Quasi-experimental, using mixed method of data collection. I note that the authors indicate that there is intention to scale up- mentioned as part of the study design. Does this mean that scale up will be explored even before developing the model (as stated in the aim) and confirming that it can achieve the desired 80% coverage?

After the preliminary assessment, how will the study sites be selected? What criteria will be used, if any?

Line 20, page 8 – sample size of 120 is for which category of study participants?

The authors indicated that the study will have formative phase (baseline), implementation phase during which information will be generated iteratively to inform the process of

building an optimal model, and then evaluation. However, the exact measurements that will be conducted and baseline and endline are not clear. I note that there will be a lot of stakeholder engagement at baseline, but it is not clear what the focus will be and how this information will be used at the endline evaluation to measure success.

During the implementation phase, the authors indicate that there will be a series of workshops with stakeholders. What is not clear is how the main goal/target of reaching 80% coverage will be measured. What data will be collected, and how, to inform progress towards achieving the 80% coverage?

VERSION 1 - AUTHOR RESPONSE

Reviewer: 1

Dr. Jayashree Gothankar, Bharati Vidyapeeth Deemed University Medical College Pune

Comments to the Author:

None.

Response: Thank you for reviewing the manuscript.

Reviewer: 2

Dr. Rebecca Nantanda, Makerere University

Comments to the Author:

Summary

This implementation research study focuses on improvements in the implementation of strategies to reduce pneumonia mortality in India, focusing on community and primary care levels. The implementation is anchored on the Childhood Pneumonia Management Guidelines of 2019 developed by the government of India as a response to the persistently high pneumonia morbidity and mortality among children less than 5 years of age. The study will use a quasi-experimental study design, and mixed (qualitative and quantitative) data collection methods will be used. It is a very important study that seeks to gather data on real-life situations and use it to develop a model that can be scaled up to other similar settings.

Response: Thank you for reviewing the manuscript and for appreciating the importance of the study.

Comments

Background

Query/Suggestion: The authors state that the pneumonia morbidity and mortality is still high, but the presumed factors, which will fully or partly be addressed by the current study are not clear.

Response: Thank you for the insightful observation. We have added few sentences on these factors in the Introduction as **"***This study specifically aims to address several of these persistent barriers—such as delayed care-seeking, low treatment compliance, inadequate provider capacity, and limited*

adherence to national pneumonia management protocols—through a locally adapted implementation strategy."

Query: It is not clear what is entailed in the Childhood Pneumonia Management Guidelines of 2019, and how different they are from all previous programs. A brief mention of how the previous programs were implemented, highlighting the gaps that will be closed by the current strategy should be mentioned.

Response: Thank you for this comment. We have now added a paragraph on Childhood pneumonia Management guideline and its difference from previous programs " Considering the substantial burden and the pressing need to intensify under-five pneumonia prevention and management efforts, *the Government of India (GoI) launched the Childhood Pneumonia Management Guidelines in 2019. (1) These guidelines represent a comprehensive shift from previous fragmented efforts by emphasizing structured case management pathways across all levels of the health system. Key features of the CPMG include a standardized management using oral amoxicillin and injectable gentamycin for pneumonia and possible serious bacterial infections (PSBI); use of pulse oximeters to assess severity, pre-referral treatment and referral mechanisms, integration of pneumonia indicators into the Health Management Information System (HMIS) and guidance for health system strengthening, including staff capacity building, community engagement, and logistics support. In pursuit of ensuring the program's visibility and long-term sustainability, the Social Awareness and Actions to Neutralize Pneumonia Successfully (SAANS) campaign was initiated, aligning with the Protect, Prevent, and Treat (PPT) interventions for childhood pneumonia. (1, 2) This annual campaign runs from November 12th (World Pneumonia Day) to February 28th ,and aims to raise awareness among caregivers and communities about pneumonia prevention, recognition, and treatment, which may eventually reduce the pneumonia burden. (1) It mobilizes Accredited Social Health Activists (ASHAs) and other front-line workers to conduct community outreach, home visits, and behavior change communication, while promoting increased demand for pneumonia services.The strategic vision revolves around the utilization of health and wellness centers (HWCs), established under the Ayushman Bharat Yojana, as central nodes for early identification, treatment, and timely referral of severe cases of under-five childhood pneumonia to tertiary healthcare facilities for life-saving interventions. (1)* "

Query: The authors also need to provide some insights into the Social Awareness and Actions to Neutralize Pneumonia Successfully (SAANS) as this seems to be only in India.

Response: Thank you for the comment. We have now added more details on SAANS " *In pursuit of ensuring the program's visibility and long-term sustainability, the Social Awareness and Actions to Neutralize Pneumonia Successfully (SAANS) campaign was initiated, aligning with the Protect, Prevent, and Treat (PPT) interventions for childhood pneumonia. (1, 2) This annual campaign runs from November 12th (World Pneumonia Day) to February 28th ,and aims to raise awareness among caregivers and communities about pneumonia prevention, recognition, and treatment, which may*

eventually reduce the pneumonia burden. (1) It mobilizes Accredited Social Health Activists (ASHAs) and other front-line workers to conduct community outreach, home visits, and behavior change communication, while promoting increased demand for pneumonia services.The strategic vision revolves around the utilization of health and wellness centers (HWCs), established under the Ayushman Bharat Yojana, as central nodes for early identification, treatment, and timely referral of severe cases of under-five childhood pneumonia to tertiary healthcare facilities for life-saving interventions. (1)"

Query: The last paragraph of the background section focuses more on what will be done, which ideally should be under the implementation process. The details of what will be done under each proposed strategy (for example augmenting all supplementary programs on pneumonia) need to be provided.

Response: Thank you for an insightful comment. We completely agree with your suggestion and now have modified the last paragraph as **"***Given this context, this implementation research was conceptualized to evaluate and optimize the delivery of the Government of India's Childhood Pneumonia Management Guidelines in a resource-constrained setting. The study focuses on achieving high population-based treatment coverage, defined as 80% or more under-five children receiving the full recommended treatment, while aligning with and strengthening existing public health infrastructure. The research also seeks to identify practical implementation barriers and develop adaptable strategies that can inform scale-up efforts in similar settings across India***.**

Query: The background and the rationale for focusing on the girl child is not provided. This needs to be included.

Response: Thank you for highlighting this. We have now incorporated the rationale in the introduction as **"***Evidence from previous studies in India indicates that gender disparities exist in healthcare-seeking behavior, with girls often receiving delayed or inadequate treatment for childhood illnesses, including pneumonia, necessitating a focused effort to promote equitable care through targeted strategies for female children.(3)*"

AIM

Study aim: The study aim is to develop a model, informed by the learnings from the implementation process. This is clear. However, the specific objective seems to be more like a desired/anticipated outcome/target rather than a research objective. This is also mentioned as a primary aim in the second last paragraph of the background section. The research question that the authors seek to answer are not stated or described.

Response: Thank you very much for highlighting these issues. We have now modified the objectives and research question as:

“Research question: How does an optimized implementation model influence the appropriate coverage of pneumonia management among under-five children, and what clinical, behavioral, and system-level factors shape treatment outcomes, adherence, and gender-related care-seeking patterns?

Primary Objective

-To develop, refine, implement, and evaluate an optimized, sustainable and scalable implementation model for improving population-based coverage of pneumonia management among under-five children, in alignment with the Government of India’s Childhood Pneumonia Management Guidelines. The model aims to achieve ≥80% treatment coverage in the target population.

Secondary Objectives

i) To assess the proportion of children who recover after seven days of treatment.
ii) To estimate the proportion of treatment failures among diagnosed pneumonia cases.
iii) To measure compliance with the recommended pneumonia treatment protocol.
iv) To determine the proportion of children who complied with hospital referral advice.
v) To document the duration and treatment details of inpatient pneumonia cases.
vi) To explore the knowledge, perceptions, and experiences of caregivers, healthcare providers, and community health workers regarding pneumonia identification, referral, and management.
vii) To identify barriers to and facilitators of equitable pneumonia treatment, particularly among girl children, in the context of the “Save the Girl Child” initiative.”

Query on Study design: Quasi-experimental, using mixed method of data collection. I note that the authors indicate that there is intention to scale up- mentioned as part of the study design. Does this mean that scale up will be explored even before developing the model (as stated in the aim) and confirming that it can achieve the desired 80% coverage?

Response: Thank you for the insightful comment. The implementation model will be iteratively refined and optimized during Phase II of the study, based on findings from the formative research and insights gathered during concurrent implementation. This iterative process will take place within a "learning block" comprising four selected Health and Wellness Centers (HWCs).

During this phase, barriers and challenges identified in the formative phase will be systematically addressed. The model will be considered **optimized** when:

- Healthcare providers can consistently recognize pneumonia signs and symptoms and manage cases in accordance with the national guidelines, with ≥80% fidelity among the suspected cases visiting the centers; and
- Treatment compliance among under-five children reaches at least 80%, who have received this treatment from the health facilties.

Once the model is optimized, it will be **scaled up** to all ten health and wellness centers in the study area. During this scale-up phase (estimated to last 7–8 months), we will continue to monitor both implementation barriers and quantitative indicators such as fidelity to the guidelines and treatment compliance.

At the conclusion of the scale-up phase, an **endline household survey** will be conducted using Probability Proportional to Size (PPS) sampling to identify 120 under-five children who had pneumonia symptoms in the past 28 days. These data will be compared with baseline measurements to assess changes in key outcome indicators i,e under-five pneumonia cases receiving management (as per the national guidelines) from the appropriate health facilities, which determines the coverage of appropriate pneumonia management.

Finally, the findings will be shared with state and district-level health authorities in Haryana. If the district government expresses interest and supports the approach, the model, having already been scaled up within the study area, may be adopted for implementation across the entire Palwal district, with continued technical support from the research team.

For better clarity we have now added all this information in the Implementation strategy section of the manuscript as: " *The model will be considered optimized when the healthcare providers can consistently recognize pneumonia signs and symptoms and manage cases in accordance with the national guidelines, with ≥80% fidelity among the suspected cases visiting the centers. Secondly, treatment compliance among under-five children reaches at least 80%, who have received this treatment from the health facilities.*

*Once the model is optimized, it will be **scaled up** to all ten health and wellness centers in the study area. During this scale-up phase (estimated to last 7–8 months), we will continue to monitor both implementation barriers and quantitative indicators such as fidelity to the guidelines and treatment compliance. At the conclusion of the scale-up phase, an **endline household survey** will be conducted using Probability Proportional to Size (PPS) sampling to identify 120 under-five children who had pneumonia symptoms in the past 28 days. These data will be compared with baseline measurements to assess changes in key outcome indicators i,e under-five pneumonia cases receiving management (as per the national guidelines) from the appropriate health facilities, which determines the coverage of appropriate pneumonia management.*

The final findings will be shared with state and district-level health authorities in Haryana. If the district government expresses interest and supports the approach, the model, having already been scaled up within the study area, may be adopted for implementation across the entire Palwal district, with continued technical support from the research team."

Query: After the preliminary assessment, how will the study sites be selected? What criteria will be used, if any?

Response: Thank you for the comment. The Health and Wellness Centers (HWCs), which serve as the primary catchment areas or study sites, will be selected based on specific criteria to ensure feasibility and readiness for implementation. The criteria include:

1. Proximity and accessibility: HWCs located within a reasonable distance (approximately 11–12 km) from the District Hospital to facilitate referrals and supervision.
2. Availability of human resources: Sites with adequate staffing necessary to deliver comprehensive primary healthcare services.
3. Basic infrastructure: HWCs with appropriate infrastructure including a functional building, essential equipment (e.g., furniture), electricity, and water supply, and that operate regularly.

Points 2 and 3 are essential prerequisites for implementing the intervention. Sites that lack these minimum requirements would require significant investment and time to upgrade, which may not be feasible within the study duration.

To enhance clarity we have now added this content to the manuscript "Methods section as, "*We will exclude HWCs that lack appropriate infrastructure and human resources, as these are basic prerequisites for implementing the intervention. Specifically, HWCs must have a functional building, essential utilities such as electricity and water supply, and basic furnishings. Additionally, the presence of adequate human resources to deliver comprehensive primary healthcare services is critical. HWCs should also be operational on a regular basis. Facilities that do not meet these criteria would require significant upgrades, which may be time-consuming and not feasible within the study period. Among the eligible facilities, preference will be given to HWCs that are reasonably accessible (within approximately 11–12 km) from the District Hospital to facilitate referral linkages and supervision."*

Query: Line 20, page 8 – sample size of 120 is for which category of study participants?

Response: Thank you. The sample size of 120 refers to under-five children who exhibit signs and symptoms of pneumonia, as reported by their primary caregivers**.**

Query: The authors indicated that the study would have formative phase (baseline), implementation phase during which information will be generated iteratively to inform the process of building an optimal model, and then evaluation. However, the exact measurements that will be conducted and baseline and endline are not clear. I note that there will be a lot of stakeholder engagement at baseline, but it is not clear what the focus will be and how this information will be used at the endline evaluation to measure success.

Response: Thank you for the observation. The primary outcome of the study is the proportion of under-five children with pneumonia who receive appropriate management (as per the Government of India's Childhood Pneumonia Management Guidelines) from appropriate sources. Appropriate sources refer to healthcare facilities and providers that adhere to these guidelines, including government facilities such as Health and Wellness Centers (HWCs), Primary Health Centers (PHCs),

Community Health Centers (CHCs), district hospitals, and qualified private clinics/hospitals. In contrast, non-appropriate sources include unlicensed practitioners, chemists, traditional or religious healers, and others who do not follow standardized case management protocols.

At baseline, approximately 120 under-five children who experienced pneumonia-like symptoms within the preceding 28 days will be identified. Their primary caregivers will be interviewed using a structured questionnaire to collect information on the source of treatment sought, treatment compliance, and clinical outcomes. Sociodemographic characteristics of the child and household will also be recorded to understand potential influencing factors.

The endline assessment will replicate the baseline methodology using the same structured questionnaire. This approach will enable comparison over time to assess shifts in care-seeking behaviour, treatment compliance, and outcomes. These findings will help us evaluate the contribution of the optimized implementation model to improving coverage of appropriate pneumonia management.

For instance, if only 10% of under-five children were receiving care from appropriate sources at baseline and this increases to 80% or more at endline, it would be considered a clear indication of the model's success in enhancing access to guideline-based pneumonia treatment.

Query: During the implementation phase, the authors indicate that there will be a series of workshops with stakeholders. What is not clear is how the main goal/target of reaching 80% coverage will be measured. What data will be collected, and how, to inform progress towards achieving the 80% coverage?

Response: Response:
Thank you for the insightful comment. We have explained the process in three different sections:

Stakeholder Engagement and Iterative Model Development:
During the implementation phase, a series of stakeholder workshops will be conducted with government healthcare authorities, community representatives, and clinicians. These workshops will serve as critical platforms for identifying challenges, co-developing mitigation strategies, and iteratively refining the implementation of the Government of India's Childhood Pneumonia Management Guidelines. Meetings will be held at regular intervals to review progress and adapt the intervention model based on real-time learnings. Once the optimized model demonstrates the ability to improve pneumonia treatment coverage to 80% or more in the initial learning block, it will be scaled up to the remaining sites. Stakeholder review meetings will continue throughout the scale-up phase to further refine the model in response to emerging barriers or implementation gaps. The final, optimized model will represent the version that has been scaled across the study area and has successfully improved pneumonia management coverage.

Measurement of the 80% Coverage Target:
The primary outcome, achieving ≥80% coverage of appropriate pneumonia management—will be

assessed using a structured quantitative household survey tool. Three cross-sectional surveys (baseline, midline, and endline) will be conducted with caregivers of under-five children who experienced pneumonia-like symptoms in the previous 28 days. These surveys will capture whether care was sought from appropriate sources and whether treatment received aligned with the national guidelines. The model will be considered successful if at least 80% of these children:

- Received care from appropriate sources (i.e., HWCs, PHCs, CHCs, or DHs), and
- Were managed according to the recommended protocols with ≥80% compliance to the prescribed treatment.

Data Collection for Monitoring Progress:
To monitor progress towards the coverage target, both quantitative and qualitative data will be collected at community and facility levels.

- Community-level quantitative data (via caregiver survey) will include:
    - Source of treatment for pneumonia
    - Time to treatment-seeking after symptom onset
    - Type of treatment received (with verification of medicines/injections where possible)
    - Compliance with treatment
    - Reported treatment outcomes
    - Out-of-pocket expenses incurred
- Community-level qualitative data will include:
    - Caregivers' knowledge of pneumonia signs and symptoms
    - Care-seeking behavior and reasons for provider preference
    - Barriers and enablers to accessing appropriate care
    - Experiences with service delivery
    - Suggestions to improve pneumonia care services
- Facility-level data will be collected by the Implementation Support Team at baseline, then bi-monthly, and finally at endline. This will include:
    - Number of suspected pneumonia cases presenting at health facilities
    - Number of cases diagnosed, treated, or referred according to guidelines
    - Number of household follow-ups conducted by ASHAs
    - Availability of essential medicines and supplies

- Status of key equipment (e.g., pulse oximeters, thermometers, weighing scales, stethoscopes)
- Human resource availability
- Training status of healthcare workers in pneumonia management

These data streams will allow ongoing tracking of both outcome-level changes (i.e., coverage and compliance) and process-level improvements (i.e., facility readiness, staff capacity), helping inform adaptive changes to ensure progress toward the 80% coverage target.

For better clarity all these information has been updated in the manuscript, Methodology section under outcomes as

“ **Outcomes**

The primary outcome of the study is to find the coverage of effective pneumonia management (i.e. management done as per the childhood pneumonia management guidelines of India) from appropriate sources. Appropriate sources refer to government and/or and private healthcare facilities like Health and Wellness Centers (HWCs), Primary Health Centers (PHCs), Community Health Centers (CHCs), district hospitals, private clinics/hospitals etc. where treatment is provided by qualified healthcare practitioner. In contrast, non-appropriate sources include unlicensed practitioners, chemists, traditional or religious healers, and others who are non-registered medical healthcare workers. The secondary outcomes are total recovered cases after seven days of treatment, treatment failure, compliance to management as recommended by healthcare providers, in-patient cases duration and treatment provided, and gender-wise distribution of diagnosed, treated, and complaint cases.

Description and tracking process of study outcomes:

The primary targeted outcome of this study is achieving ≥80% coverage of appropriate pneumonia management and will be assessed using a structured quantitative household survey tool. Three cross-sectional surveys (baseline, midline, and endline) will be conducted with caregivers of under-five children who experienced pneumonia-like symptoms in the previous 28 days. These surveys will capture whether care was sought from appropriate sources and whether treatment received aligned with the national guidelines. The success of this research will be when at least 80% of these children:

- *Received care from appropriate sources and*
- *Were managed according to the recommended national protocols with ≥80% compliance to the prescribed treatment.*

Data Collection for Monitoring Progress:

To monitor progress towards the coverage target, both quantitative and qualitative data will be collected at community and facility levels.

- *Community-level quantitative data (via household cross-sectional survey) will include:*
  - *Source of treatment for pneumonia*
  - *Time to treatment-seeking after symptom onset*
  - *Type of treatment received (with verification of medicines/injections where possible)*
  - *Compliance with treatment*
  - *Reported treatment outcomes*
  - *Out-of-pocket expenses incurred*
- *Community-level qualitative data will include:*
  - *Caregivers' knowledge of pneumonia signs and symptoms*
  - *Care-seeking behavior and reasons for provider preference*
  - *Barriers and enablers to accessing appropriate care*
  - *Experiences with service delivery*
  - *Suggestions to improve pneumonia care services*
- *Facility-level data will be collected at baseline (Phase-I), then bi-monthly(Phase-II and III), and finally at endline(endline survey) . This will include:*
  - *Number of suspected pneumonia cases presenting at health facilities*
  - *Number of cases diagnosed, treated, or referred according to guidelines*
  - *Number of household (diagnosed cases of pneumonia) follow-ups conducted by ASHAs*
  - *Availability of essential medicines and supplies*
  - *Status of key equipment (e.g., pulse oximeters, thermometers, weighing scales, stethoscopes)*
  - *Human resource availability*
  - *Training status of healthcare workers in pneumonia management*

These data streams will allow ongoing tracking of both outcome-level changes (i.e., coverage and compliance) and process-level improvements (i.e., facility readiness, staff capacity), helping inform adaptive changes to ensure progress toward the final targeted outcome."
